# Effects of Population Bottleneck and Balancing Selection on the Chinese Alligator Are Revealed by Locus-Specific Characterization of MHC Genes

**DOI:** 10.1038/s41598-017-05640-2

**Published:** 2017-07-17

**Authors:** Teng Zhai, Hai-Qiong Yang, Rui-Can Zhang, Li-Ming Fang, Guo-Heng Zhong, Sheng-Guo Fang

**Affiliations:** 1 The Key Laboratory of Conservation Biology for Endangered Wildlife of the Ministry of Education, and State Conservation Center for Gene Resources of Endangered Wildlife, College of Life Sciences, Zhejiang University, Hangzhou, Zhejiang 310058 China; 2 Changxing Yinjiabian Chinese Alligator Nature Reserve, Changxing, Zhejiang 313100 China

## Abstract

Chinese alligator (*Alligator sinensis*) is an endangered freshwater crocodilian endemic to China, which experienced a severe bottleneck about 30 years ago. In this study, we developed locus-specific primers to investigate the polymorphism of 3 major histocompatibility complex (MHC) loci in 3 Chinese alligator populations, in combination with 6 neutral microsatellite markers as a contrast. We found the genetic trace for the bottleneck effect on the endangered Chinese alligator: the low allelic diversity (2 alleles at each locus), the low nucleotide substitution rate (no more than 0.009) at all sites, the deviation from Hardy-Weinberg Equilibrium/heterozygote deficiency, and the significant Tajima’s D values, indicating the MHC class I and class II loci being at different stages of bottleneck. We also obtained 3 pieces of evidence for balancing selection on this severely bottlenecked reptile: an obvious excess of nonsynonymous substitutions over synonymous at the antigen-binding positions, the mean synonymous substitution rate of MHC exons significantly higher than mean nucleotide substitution rate of introns, and the differentiation coefficient *F*_ST_ of MHC loci significantly lower than that of microsatellite loci. Consequently, we emphasize that the Chinese alligator holds a pretty low adaptive ability and requires scientific conservation strategies to ensure the long-term population development.

## Introduction

The Chinese alligator (*Alligator sinensis*) is a very ancient species that had once been pushed to the fringe of extinction by massive hunting, territory deprivation and environment deterioration^1^. In the 1960s, the wild Chinese alligators become very scarce, and the complicated environments of wetlands and swamps made them even harder to be spotted^2^. The population of Chinese alligator didn’t stop dropping until 1979 when Changxing Yinjiabian Chinese Alligator Nature Reserve (CYCANR) and Anhui Research Center for Chinese Alligator Reproduction (ARCCAR) were founded to conserve this species, starting with 7 and 212 founder alligators respectively. As of today, there are more than 8,000 captive Chinese alligators at both reserve sites, making the ARCCAR and CYCNAR populations not only the last but also the largest captive populations in China. On the other hand, the number of the Chinese alligators in the wild is dropping 20% every year and the current number is predicted to be lower than 120^2, 3^.

Judging from the population size alone, the Chinese alligators seem to be recovering quite well from the bottleneck event 30 years ago, and previous studies on the major histocompatibility complex (MHC) genes didn’t raise alarms on any of the repercussions of the bottleneck effect. The MHC genes are not only the most functionally polymorphic genes in vertebrates, but also mediate immune responses, thus making them good adaptive markers to assess the evolutionary potential of fighting pathogens^4^. Previous studies on the Chinese alligator MHC II genes had shown great polymorphism in exon 2, exon 3 and even intron 2 sequences^5–8^. After the publication of crocodile, alligator and gharial genomes^9–11^, the structure and polymorphism of the MHC genes in the crocodilians has been massively annotated and studied^12, 13^. However, all the above-mentioned studies have been using universal primers to investigate the Crocodylia MHC genes. Considering the evidence of MHC gene duplication^12, 14^, it’s possible that the cross-locus amplification would cause sequences from different loci being regarded as alleles from a single locus, thus distorting the true intra-locus alignment and elevating the rate of non-synonymous (*d*_N_) over synonymous (*d*_S_) substitutions. Hence, the true level of genetic diversity at single MHC genes of the Crocodylia is still pending for exploration.

In our previous studies on the Chinese alligator, He *et al*. constructed a bacterial artificial chromosome (BAC) library that contains 6.8-fold genome equivalents^15^. Ye *et al*. constructed several contigs containing MHC genes based on the BAC library^16^. Wan *et al*. published the Chinese alligator genome as 2.3 Gb in size, and annotated over 22,200 genes^16^. These works enable us to design locus-specific primers to characterize MHC genes, hoping to investigate whether bottleneck event continues to impact the Chinese alligator population at the single gene level even after an exponential population growth, to unveil the actual polymorphism of a single MHC gene, and to elaborate the evolutionary forces that influence MHC genes.

## Results

### BLAST results and Genotyping of MHC genes

By BLASTing the BAC-end and known MHC sequences to the *A. sinensis* genome and predicting potential MHC genes, we found 3 MHC loci with intact coding sequences and gene structures. The 3 MHC loci can be pinpointed to different *A. sinensis* genome scaffolds: scaffold1303_1 (NCBI accession number: NW_005842753), scaffold364_1 (NCBI accession number: NW_005842546) and scaffold184_1 (NCBI accession number: NW_005842983). The 3 MHC loci were also verified to be from 3 BAC clones that had no overlap sequences, suggesting that they were independent loci. The BAC clones 1327C2 and 20A2 contained 2 MHC I loci and the BAC clone 1085A9 contained 1 MHC II locus; these MHC loci were accordingly named I1327, I20 and Beta1085, respectively.

**Figure 1:**
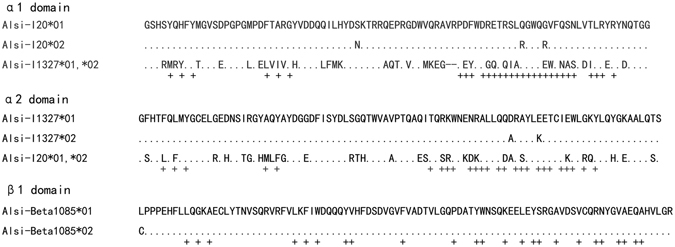
Amino acid sequence alignment of 3 MHC loci. Dots represent identical amino acids to the top variant and crosses under the alignment depict putative antigen binding sites.

The single strand conformation polymorphism (SSCP) results of these loci were all di-morphic: 2 alleles at each locus, which were confirmed by the sequencing results. Nonetheless, the gene I1321 is actually mono-morphic at the functional level due to the same amino acid sequence of the 2 alleles (Fig. 1). No more than two sequences were present in each animal, demonstrating no cross-locus amplification in our study, and none of the sequences showed deletions, insertions, or stop codons, showing the accurate genotyping at single functional MHC genes. In total, we obtained 6 nucleotide acid alleles but 5 amino acid alleles from the 3 MHC loci using the locus-specific genotyping primers (Fig. 1), suggesting an extremely low level of adaptive genetic variation in the endangered Chinese alligator.

### Allelic distribution, heterozygosity and Hardy-Weinberg Equilibrium test

The sequence analysis exhibits a quite low level of nucleotide diversity (0.0025—0.0128) in all the MHC loci (Table 1). These results were unlike previous MHC studies in *A. sinensis* ^5, 6^, likely because of their use of degenerate primers that are capable of amplifying more than one MHC locus. However, our results coincided with results from Wan *et al*., which revealed low SNP heterozygosity throughout the *A. sinensis* genome^11^.

**Table 1:** Genetic diversity at the MHC loci.

Locus	*N*_A_	Nucleotide length (bp)			*Hd*		*π*	
		Exon 2	Intron 2	Exon 3	Exon	Intron	Exon	Intron
I1327	2	261	15135	276	0.444 ± 0.064	0.467 ± 0.017	0.25%	0.20%
I20	2	267	914; 925	276	0.457 ± 0.055	0.356 ± 0.025	0.34%	0.20%
Beta1085	2	282	631; 632	—	1.000 ± 0.052	0.182 ± 0.021	0.94%	0.06%

Note: *N*_A_, the number of alleles; *Hd*, haplotype diversity; *π*, nucleotide diversity.

Although varying with loci, the allelic distribution patterns of the same MHC gene are similar among populations (Table 2). The Hardy-Weinberg Equilibrium (*HWE*) test results show a severe heterozygote deficiency at the Beta1085 locus in ZJ and AH populations. Differently, the Tajima’s D test results are strange (Table 2): below 0 value at MHC class II locus (significant in ZJ population), and above 0 value at both MHC class I loci (significant in ZJ and AH population). The different Tajima’s D values between the class I and II MHC loci might indicate different stages of bottleneck or different selection pressures.

**Table 2:** Genetic diversity of MHC in 3 Chinese alligator populations.

Population	Locus	Allele 01	Allele 02	*H*_O_	*H*_E_	*P-value of HWE*	Tajima’s D test
ZJ	I1327	**0.73**	0.27	0.469	0.396	0.396	1.711
AH	I1327	**0.69**	0.31	0.375	0.437	0.441	2.083*
USA	I1327	**0.70**	0.30	0.200	0.442	0.132	1.518
ZJ	I20	0.36	**0.64**	0.531	0.468	0.699	2.611*
AH	I20	0.22	**0.78**	0.375	0.347	1.000	1.383
USA	I20	0.10	**0.90**	0.200	0.189	1.000	0.949
ZJ	Beta1085	**0.98**	0.02	0.031	0.092	0.015*	−1.953*
AH	Beta1085	**0.91**	0.09	0.125	0.231	0.008*	−1.524
USA	Beta1085	**1.00**	0.00	—	—	—	—

Note: the predominant alleles are in bold; * indicates *P* < 0.05; *H*_O_ and *H*_E_ are observed and expected heterozygosities, respectively.

The repercussions of bottleneck effects can also be observed at the microsatellite loci (Table 3), where allelic diversity is very low (2.5 alleles per locus) in all 3 populations. Despite the higher *H*_O_ than *H*_E_ at several loci, this phenomenon should be attributed to the selection of microsatellite markers with high-resolution power in the study of Ma^17^.

**Table 3:** Genetic diversity of microsatellite markers in 3 Chinese alligator populations.

**Locus**	**ZJ (n = 32)**			**AH (n = 32)**			**USA (n = 10)**		
	**N**_**A**_	**H**_**O**_	**H**_**E**_	**N**_**A**_	**H**_**O**_	**H**_**E**_	**N**_**A**_	**H**_**O**_	**H**_**E**_
CXA-142	2	0.844*	0.503	2	0.536	0.399	2	0.800	0.505
CXA-41	2	0.688*	0.476	2	0.393	0.431	2	0.111	0.111
CXA-6	2	0.500	0.411	2	0.615	0.507	2	0.700	0.479
CXA-9	3	0.250*	0.405	3	0.654	0.578	2	1.000*	0.526
CXA-34	3	0.500*	0.608	3	0.786*	0.590	3	0.900*	0.679
CXA-43	3	0.531*	0.587	4	0.536	0.543	2	0.700	0.521
Mean	2.5	0.552	0.498	2.667	0.587	0.508	2.167	0.702	0.470
SD	0.548	0.200	0.086	0.816	0.132	0.078	0.408	0.312	0.190

Note: *N*_A_, number of alleles; *H*_O_, observed heterozygosity; *H*_E_, expected heterozytosity; * shows significant deviation from HWE (*P* < 0.05).

### **Calculation of*d***_**N**_**and*d***_**S**_**substitutions**

The *d*_N_/*d*_S_ ratio can provide useful information on the degree of selective pressure acting on a protein-coding gene^18^. The *d*_N_/*d*_S_ > 1 implies positive selection; *d*_N_/*d*_S_ < 1 implies purifying (negative) selection; and *d*_N_/*d*_S_ = 1 indicates neutral (i.e. no) selection. As we can see in Table 4, the Chinese alligator MHC exons exhibit extremely low *d*_N_ and *d*_S_ values due to the low allelic and nucleotide diversity, and the Z-tests show no significant P value at any locus. The following trends, however, can be observed: at the MHC class I loci, the *d*_N_/*d*_S_ ratios all exceed 1 at antigen binding sites (ABS), showing the sign of positive selection. Nonetheless, at the MHC class II locus, there is no nucleotide substitution at ABS positions (Fig. 1), thus indicating purifying selection. When the *d*_N_/*d*_S_ values were compared between the ABS and non-ABS sites, the MHC class I loci produced a larger ratio at ABSs than at non-ABSs while the MHC class II locus showed *d*_S_ at non-ABSs in spite of zero *d*_N_ changes (Table 4), indicating balancing selection at play in the Chinese alligator.

**Table 4:** The *d*_N_/*d*_S_ ratio of 3 MHC loci in 3 Chinese alligator populations.

Locus	Position	*N*	ZJ			AH			USA		
			*d*_N_	*d*_S_	*d*_N_/*d*_S_	*d*_N_	*d*_S_	*d*_N_/*d*_S_	*d*_N_	*d*_S_	*d*_N_/*d*_S_
I1327	ABS	56	0.008 ± 0.005	0.005 ± 0.006	1.60	0.008 ± 0.006	0.006 ± 0.006	1.33	0.009 ± 0.006	0.006 ± 0.006	1.50
	non-ABS	125	0	0	0	0	0	0	0	0	0
	All	181	0.002 ± 0.002	0.002 ± 0.002	1.00	0.003 ± 0.002	0.002 ± 0.002	1.50	0.003 ± 0.002	0.002 ± 0.002	1.50
	ABS	56	0.007 ± 0.005	0	∞	0.005 ± 0.004	0	∞	0.003 ± 0.002	0	∞
I20	non-ABS	125	0.002 ± 0.002	0.005 ± 0.006	0.40	0.001 ± 0.001	0.004 ± 0.004	0.25	0.001 ± 0.001	0.002 ± 0.002	0.50
	All	181	0.003 ± 0.002	0.004 ± 0.004	0.75	0.003 ± 0.001	0.003 ± 0.003	1.00	0.001 ± 0.001	0.002 ± 0.001	0.50
	ABS	49	0	0	0	0	0	0	—	—	—
Beta1085	non-ABS	139	0	0.001 ± 0.001	0.00	0.002 ± 0.002	0.005 ± 0.004	0.40	—	—	—
	All	188	0	0.001 ± 0.001	0.00	0.001 ± 0.001	0.004 ± 0.003	0.25	—	—	—

Note: the *d*_N_/*d*_S_ ratios are calculated separately for antigen-binding site (ABS), non-ABS and all sites. Codon numbers (*N*) of the sites are shown. In the USA population, Beta1085 locus has only one allele, thus no *d*_N_ and *d*_S_ values are calculated.

### Comparison of nucleotide substitution between exon and intron

**Figure 2:**
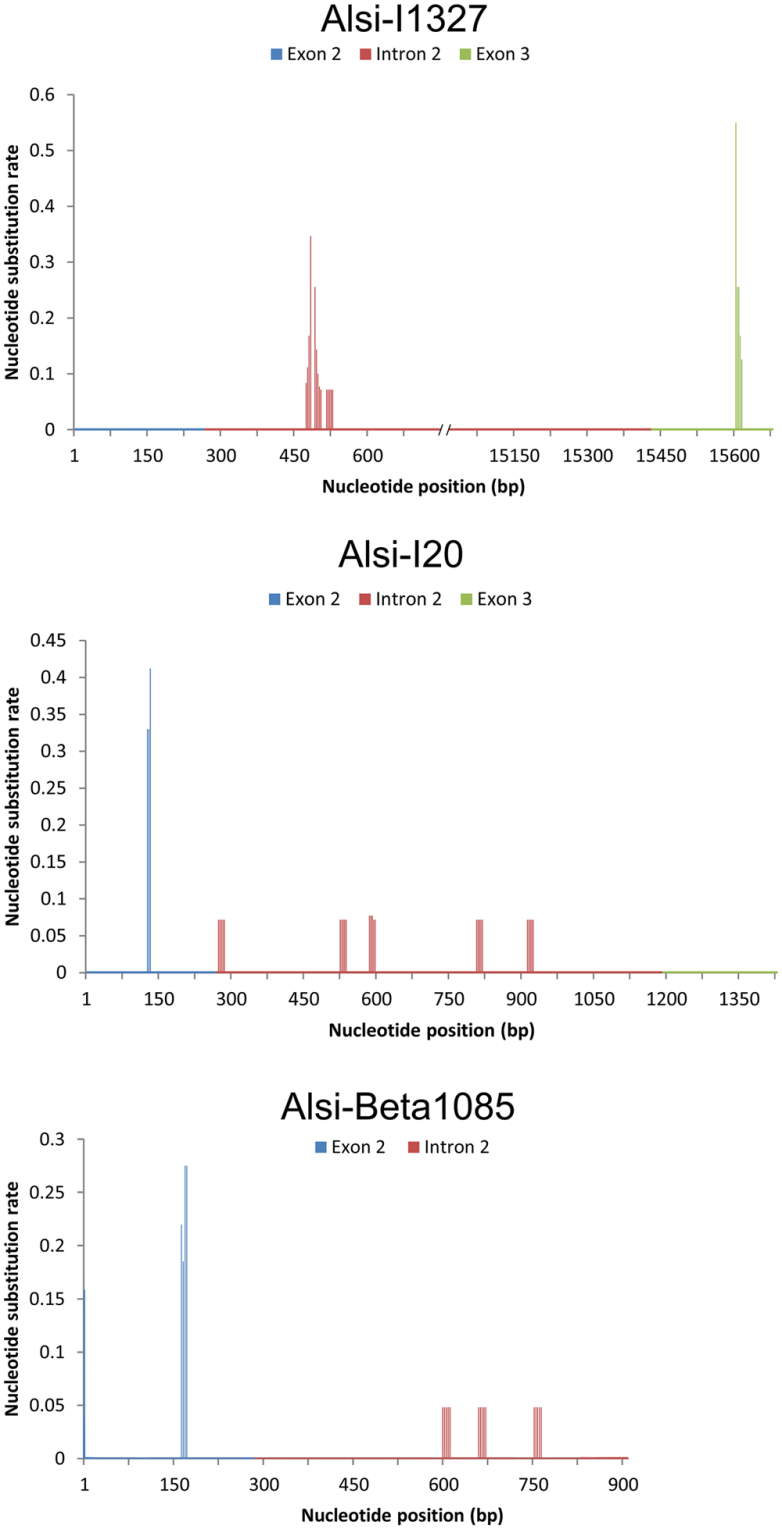
A plot of *d*_S_ in exon 2 (and exon 3 at class I genes) versus *d* in intron2.

All 3 Chinese alligator populations shared the same allele sequences in all 3 MHC loci, and the only differences were the allelic frequencies at each locus. Thus, we computed nucleotide substitution on the basis of these unique allele sequences and without consideration of allelic frequency and population identity. We calculated the mean *d*_S_ in exons and the mean number of nucleotide substitutions per site (*d*) in introns of the MHC class I and II genes, and plotted them against the nucleotide position (Fig. 2). The results show a much higher substitution rate in the exon 2 and 3 region than in the intron 2 at all MHC loci, which means the introns are younger than exons, suggesting the exons being constantly maintained by balancing selection for ages.

### Comparison of differentiation degree between the MHC and microsatellite loci

Since the MHC genes are constantly maintained by balancing selection, even in different populations the MHC allelic frequencies will be pretty similar due to the influence of balancing selection. The microsatellite loci, however, will take more diverted evolutionary paths under the pressure of genetic drift. We tested pairwise *F*_ST_ of the 3 Chinese alligator populations at both MHC and microsatellite loci, and found significant population differentiation among 3 populations at the microsatellite loci (*P* < 0.05) with no differentiation at the MHC loci (Table 5). This contrast indicates that even though severely damaged by the bottleneck effect, the Chinese alligator MHC loci are still influenced by balancing selection, i.e. MHC genes maintain more similar alleles than neutral markers do.

**Table 5:** Pairwise *F*_ST_ test among 3 Chinese alligator populations.

	ZJ	AH	USA
ZJ	—	0.01449	0.04720
AH	0.08424*	—	0.00670
USA	0.08990*	0.06212*	—

Note: the numbers on the left side below the diagonal and on the right side above the diagonal are the *F*_st_ values of microsatellite and MHC loci, respectively. * indicates *P* < 0.05.

## Discussion

Normally, when a population encounters bottleneck events, rare alleles are more likely to be lost than the common alleles, and positive Tajima’s D values are expected; when population expands, the segregating sites accumulate at the rare frequencies, thus leading to negative Tajima’s D values^19^. In the Chinese alligator’s case, the bottleneck effect 30 years ago was too severe for the population to attain ample rare alleles with low frequencies, as evidently shown by the di-morphism MHC loci in this study. Therefore, the positive Tajima’s D value at the MHC class I loci should be a normal case for the Chinese alligator population. The negative Tajima’s D value at the MHC class II locus, however, could indicate the ongoing population expansion or purifying selection, whose trace can also be found in the sequence alignments (Fig. 1) and *d*_N_, *d*_S_ results (Table 4); the MHC class I loci have non-synonymous nucleotide substitutions at and next to the ABS sites whereas the MHC class II locus has no nucleotide substitution at all at any ABS sites.

As our abovementioned results have shown, even after 30 years, the bottleneck effect still influences the MHC genes of the Chinese alligator. That’s why conventional methods – such as *d*_N_/*d*_S_ values – function poorly in detecting balancing selection. The significant *d*_N_/*d*_S_ values in previous studies^5, 7^ are more likely caused by their usage of universal primers, as Miller and Lambert pointed out, the usage of universal primers could produce a lower than expected *d*_S_ value, leading an elevated *d*_N_/*d*_S_ ratio and thus giving “anomalous” results^20^.

Therefore, we need to compare the theoretically balancing-selection-influenced sites to those non-balancing-selection-influenced sites to reveal the presence of balancing selection. The comparison between exons and introns would be an effective tool. Hughes and Yeager^21, 22^ discovered that in most genes, the mean number of nucleotide substitutions per site (*d*) in introns and the mean *d*_S_ in exons are usually about equal – for their mutations are both selectively neutral to a gene – except when it comes to the MHC genes, the mean *d*_S_ in exons is always much higher than the mean *d* in introns. They explained that exons in the MHC genes are very ancient because they have been maintained by balancing selection for a very long time, while the mutations in introns are controlled by recombination and genetic drift. When an ancient intron polymorphism is lost due to recombination or drift, it will be selectively neutral to the MHC gene. As time goes by, introns in the MHC genes will become evolutionarily younger on average than are the exons constantly maintained by balancing selection. The much older exons will consequently possess a much higher *d*_S_ than that of the younger introns, hence mean *d*_S_ > mean *d* in MHC genes^22^. Our findings in this study support Hughes and Yeager’s theory.

The environmental factors could be another cause of the similar MHC genes among populations. Both AH and ZJ populations are intensively managed as captive populations, and their habitats are modified to natural wetlands surrounded by farming countryside. The isolated habitats may provide an environment with low level exposure to pathogens, and cause the MHC genes lack motivations to change; similar situations happen to many other species^20, 23–25^. Even if the Chinese alligator populations are sustainable at present, they would still be susceptible to new pathogens when reintroduced to the wild in the future. While the balancing selection is working on the MHC genes, proper conservation strategy should be devised to protect this endangered species.

## Materials and Methods

### Animal experiment ethics statement

All experiments were carried out in accordance with the guidelines issued by the Ethical Committee of Laboratory Animal of Zhejiang University, and all experimental protocols were approved by the Ethical Committee of Laboratory Animal of Zhejiang University.

### Sampling

The *A. sinensis* samples (see Supplementary Table S1) came from 3 captive populations, kindly provided by CYCANR (the Zhejiang population, acronym ZJ), ARCCAR (the Anhui population, acronym AH) and the Rockefeller Wildlife Refuge (the USA population, acronym USA). Although there are more than 8,000 Chinese alligators in the ZJ and AH populations, most of them are still juvenile while several hundreds of Chinese alligators are breeding adults. In this study, all sample donors were adult male/female Chinese alligators that were randomly captured, and we believe they can represent the species’ natural range. The blood samples were taken during routine medical examinations. DNA was extracted using a traditional phenol-chloroform method^26^.

### Genome BLAST and primer design

We screened the BAC clones using the universal MHC primers of Ye *et al*.^16^ and pinpointed each MHC gene to their corresponding genome scaffolds (requiring >95% in both gene coverage and sequence similarity) by BLASTing the end sequences of the target BACs and the known MHC sequence against the *A. sinensis* genome^11^ using default parameters of a local BLAST tool downloaded from NCBI (ftp://ftp.ncbi.nlm.nih.gov/blast/executables/blast+/LATEST/). Then, we used Augustus (http://augustus.gobics.de/) to predict exon positions for each MHC sequence, and manually translated each exon to amino acid sequences in order to search for MHC loci with intact coding sequences and gene structures. All the MHC genes from the BAC and the genome were manually checked for gene structural integrity to rule out pseudogenes. The MHC genes with a complete gene structure were selected for subsequent studies. We gave prefix ‘*Alsi*’ to all the MHC genes.

We then designed locus-specific primers to amplify the antigen binding domains of the MHC loci (exons 2 and 3 for the MHC class I, and exon 2 for the MHC class II). Intron 2 sequences were also amplified and sequenced to ensure the correctness of genotyping as well as to be a useful comparison material (see Supplementary Table S2). Primers were designed using Primer Premier 5 (http://www.premierbiosoft.com/), and the primers’ binding positions are illustrated in Supplementary Fig. S1. We performed pre-experiments to investigate the polymorphism of each locus using 10 randomly selected blood samples in each population. We chose the polymorphic loci to genotype all the samples in 3 populations. Six microsatellite markers from our former works were also used as a neutral contrast^17^.

### PCR amplification and genotyping

The PCR amplifications were performed in a 10 μl reaction system containing 1 L of template DNA (30–50 ng/µL), 0.5 U rTaq DNA polymerase (TaKaRa), 1 µL of 10 × ExTaq buffer (TaKaRa), 1 µL of 2.5 mmol/L dNTPs, 0.2 µL of each primer (diluted to 10 mol/µL), and 6.5 µL of double-distilled water. The PCR was programmed as follows: initial denaturation at 95 °C for 5 min, followed by 35 cycles of 95 °C for 30 s, 63 °C for 30 s, and extension at 72 °C for 30 s, with a final extension of 72 °C for 5 min.

We adopted Single-Strand Conformation Polymorphism - heteroduplex (SSCP-HD) techniques^27^ to genotype each individual and screen 3 Chinese alligator populations in order to examine the level of adaptive genetic variation in this endangered reptile. In the SSCP-HD analysis, we added 5 μl of 2X loading buffer (95% formamide, 10 mm NaOH, 0.25% bromophenol blue, 0.25% xylene cyanol) for each 10 μl of PCR product. And the mixture was denatured at 95 °C for 5 min, and swiftly transferred onto ice for cooling down. Then, the PCR products were run in an acrylamide gel consisting of 12% 37.5:1 acrylamide/bisacrylamide with 2.5% crosslinking, and sequences were separated in 0.5 × TBE running buffer at 16 °C by 150 V for 6.5 h on the Decode System (Bio-Rad). Finally, the SSCP gel was fixed in 10% acetic acid for 30 min, washed with dH2O, and achieved silver staining pictures. We repeated the PCR-SSCP process for 3 times at each locus to make sure the banding pattern was stable and consistent, and obtained the allele sequences by sequencing the homozygous individuals with unique banding patterns in BGI, Shanghai. Microsatellite loci were amplified and genotyped as described by Ma^17^.

### Data analyses

We used Lasergene 7 (DNASTAR) for nucleotide sequence editing and Mega 5^28^ for sequence alignment. We used DnaSP^29^ to calculate the haplotype diversity (*Hd*), the nucleotide diversity (*π*), and Tajima’s D values. The non-synonymous (*d*_N_) and synonymous (*d*_S_) substitution rates of the MHC exons were calculated using Mega 5, and we used K-estimator^30^ with Kimura-2p method to compute nucleotide substitution rate of intron 2 (*d*) as well as the *d*_S_ of exon 2 and exon 3, which were then plotted against nucleotide position using a sliding window size of 15 base pairs and steps of 3 base pairs. We used Kaufman’s study^31^ as a reference to annotate antigen-binding sites.

Allelic frequency, observed heterozygosity (*H*_O_), expected heterozygosity (*H*_E_) and *HWE*, as well as pairwise *F*_ST_ among 3 populations were calculated in Arlequin 3.5^32^.

### Data availability statement

The genomic sequences of MHC genes are collected from the scaffolds 1303_1 (GenBank accession number: NW_005842753), 364_1 (NW_005842546) and 184_1 (NW_005842983) of the Chinese alligator genome (GCA_000455745.1). The new nucleotide acid sequences obtained in this study are available in the supplementary Data S1.

## Electronic supplementary material

Supplementary Information
