# Supplementary material for: Effects of Population Bottleneck and Balancing Selection on the Chinese Alligator Are Revealed by Locus-Specific Characterization of MHC Genes
Source: Sci Rep. 2017 Jul 17;7:5549. doi: 10.1038/s41598-017-05640-2 (PMC5514082; doi:10.1038/s41598-017-05640-2)
Supplement: Supplementary file 1 — Supplementary Information [file 41598_2017_5640_MOESM1_ESM.doc]

**Effects of Population Bottleneck and Balancing Selection on the Chinese Alligator Are Revealed by Locus-Specific Characterization of MHC Genes**

Teng Zhai ^1^, Hai-Qiong Yang^1^, Rui-Can Zhang^1^, Li-Ming Fang ^2^, Guo-Heng Zhong^1^, and Sheng-Guo Fang ^1*^

^1^ The Key Laboratory of Conservation Biology for Endangered Wildlife of the Ministry of Education, and State Conservation Center for Gene Resources of Endangered Wildlife, College of Life Sciences, Zhejiang University, Hangzhou, Zhejiang 310058, China

^2^ Changxing Yinjiabian Chinese Alligator Nature Reserve, Changxing, Zhejiang 313100, China

^*^ Corresponding author (Email: sgfanglab@zju.edu.cn).

Table S1. Blood sample Information

| Population | Source | Number |
| --- | --- | --- |
| AH | ARCCAR | 32 |
| ZJ | CYCANR | 32 |
| USA | CYCANR | 10 |
|  |  | |

Table S2. Primer Information

| Locus | Primer name | Primer sequence (5'-3') | Product length (bp) | Ta (°C ) |
| --- | --- | --- | --- | --- |
| I1327 exon 2 | I1327E2F | GTGGGTAAGCGGCCCTCTGC | 366 | 66 |
|  | I1327E2R | CTGCCCTCCCTGCCCCCCCCG |  |  |
| I1327 exon 3 | I1327E3F | AGGGGCCTGGATCTGTGTTTG | 342 | 63 |
|  | I1327E3R | CCTGGTTAGTGCTGCCGTTA |  |  |
| I20 exon 2 | I20E2F | GCCCGCTAGTGCTGACCATC | 323 | 63 |
|  | I20E2R | CCTGCTCACACCTGCCTGCTA |  |  |
| I20 exon 3 | I20E3F | TCAGCCTGGAAGCCCTCAA | 417 | 66 |
|  | I20E3R | GGCGGGAATTGCCTGGGT |  |  |
| Beta1085 exon 2 | Beta1085E2F | GTGCTGTGGCTGGGGAGGTT | 364 | 63 |
|  | Beta1085E2R | CAACAAAGACCCCAGGAAT |  |  |
| I1327 exon 2 and flanking intron 2 | I1327E2i2F | GCAAGAGTGGTTCAATGCGAGT | 731 | 62 |
|  | I1327E2i2R | TTCCCAGCCCCTTCCCTAG |  |  |
| I1327 exon 3 and flanking intron 2 | I1327i2E3F | TAGCAACTTCCCTGCCCTCACT | 722 | 60 |
|  | I1327i2E3R | GAAAGTATGAAACCCTGAAACAAAC |  |  |
| I20 exon 2 ~ intron 2 ~ exon 3 | I20E2F | GCCCGCTAGTGCTGACCATC | 1468 | 66 |
|  | I20E3R | GGCGGGAATTGCCTGGGT |  |  |
| Beta1085 exon 2 ~ intron 2 | Beta1085E2F | GTGCTGTGGCTGGGGAGGTT | 982 | 64 |
|  | Beta1085I2R | GACTTTCACCTTGGGCTTCGC |  |  |
|  |  |  |  |  |

Figure S1. Primer positions


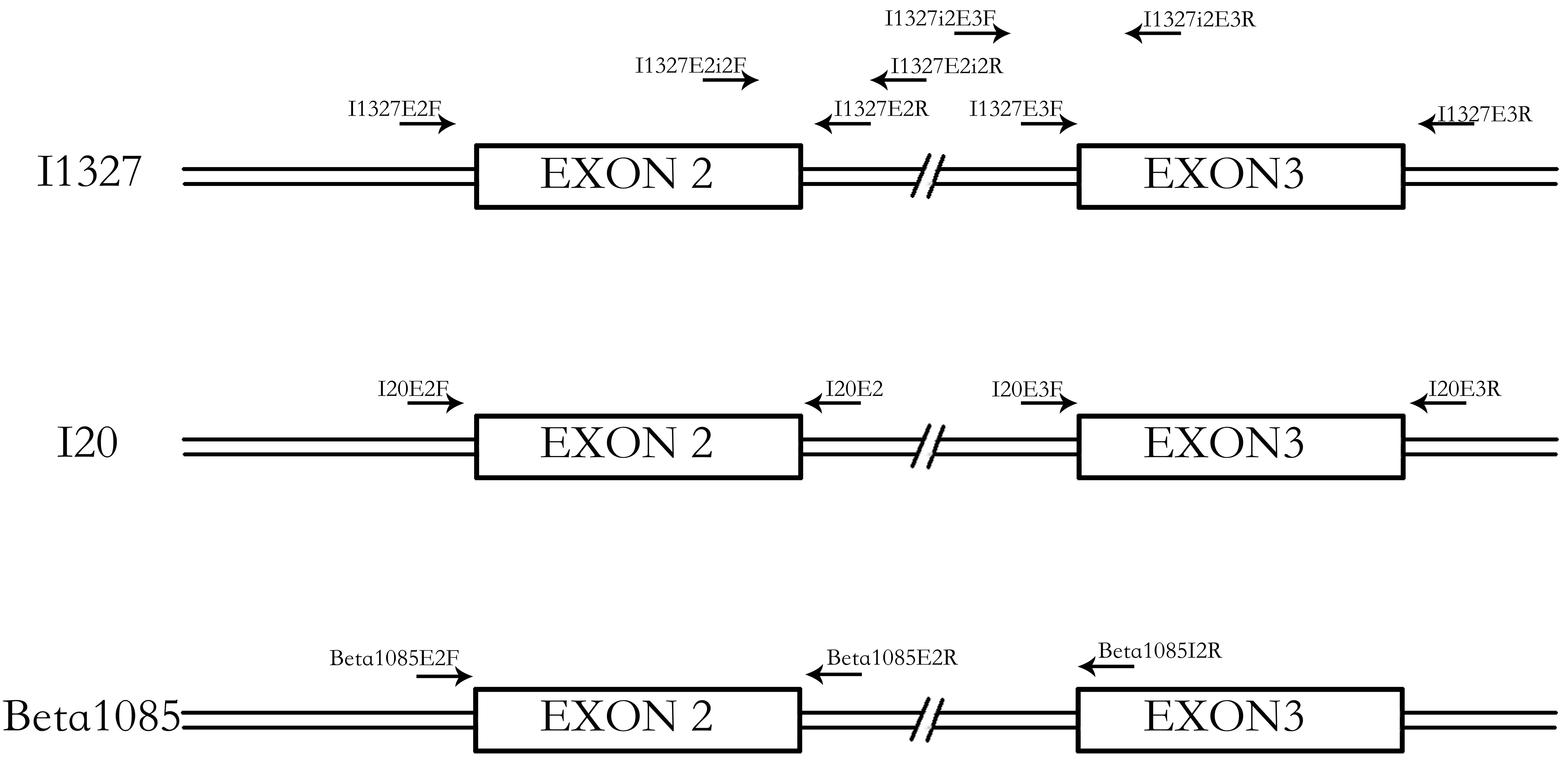


Data S1

>I1327-E2*01

GGCTCGCACCGAATGCGTTATTTCTATACGGGGGTGTCGGAGCCTGGCCCGGGGCTGCCGGAGCTTGTCATAGTCGGGCACGTGGATGACCAGCTCTTCATGAAGTATGACAGCAAGACCAGGAGGGCTCAGACCCGTGTGGACTGGATGAAGGAGGGCCCAGAGTACTGGGATGGCCAGACCCAGATCGCGCAGGGCTGGCAAGAGTGGTTCAATGCGAGTTTGGACATTCTGCGAGAGCGCTACGACCAGACTGGCGGT

>I1327-E3*01

GGGTTTCATACTTTCCAGCTCATGTACGGCTGTGAGCTTGGTGAAGACAACAGCATTCGAGGCTATGCGCAGTATGCGTATGACGGAGGAGACTTCATCAGCTATGACCTGAGCGGGCAAACCTGGGTGGCAGTCCCAACACAGGCCCAGATCACCCAGCGCAAGTGGAATGAGAATAGGGCCTTACTTCAGCAAGACAGAGCCTACCTGGAGGAGACCTGCATCGAGTGGCTGGGGAAGTACCTACAGTACGGGAAGGCAGCGCTGCAGACCAGT

>I1327-E3*02

GGGTTTCATACTTTCCAGCTCATGTACGGCTGTGAGCTTGGTGAAGACAACAGCATTCGAGGCTATGCGCAGTATGCGTATGACGGAGGAGACTTCATCAGCTATGACCTGAGCGGGCAAACCTGGGTGGCAGTCCCAACACAGGCCCAGATCACCCAGCGCAAGTGGAATGAGAATAGGGCCTTACTTCAGCAAGCGAGAGCCTACCTGAAGGAGACCTGCATCGAGTGGCTGGGGAAGTACCTACAGTACGGGAAGGCAGCGCTGCAGACCAGT

>I20-E2*01

GGCTCCCACAGCTACCAGCATTTCTACATGGGGGTGTCAGACCCCGGCCCAGGCATGCCCGACTTCACTGCCAGGGGCTATGTGGACGACCAGCAAATTCTCCACTATGACAGCAAGACACGGAGACAGGAGCCACGCGGAGACTGGGTGCAGAGAGCCGTCAGACCAGACTTCTGGGACAGGGAAACCAGAAGCTTGCAGGGCTGGCAGGGGGTATTTCAATCAAACCTGGTCACCCTGCGGTATCGCTACAACCAGACCGGTGGT

>I20-E2*02

GGCTCCCACAGCTACCAGCATTTCTACATGGGGGTGTCAGACCCCGGCCCAGGCATGCCCGACTTCACTGCCAGGGGCTATGTGGACGACCAGCAAATTCTCCACTATGACAACAAGACACGGAGACAGGAGCCGCGCGGAGACTGGGTGCAGAGAGCCGTCAGACCAGACTTCTGGGACAGGGAAACCAGAAGCTTGCGGGGCTGGCAGCGGGTATTTCAATCAAACCTGGTCACCCTGCGGTATCGCTACAACCAGACCGGTGGT

>I20-E3*01

GGGTCTCACACTCTCCAGTTCATGTACGGCTGTGAGCTCCGTGAACACAACAGCACTGGAGGCCACATGCTGTTTGGCTATGATGGGGAAGACTTCATCAGCTATGACCTGAGAACACACACCTGGGTAGCAGCCCCGACACAGGCTGAGAGCACCCAGAGCAGGTGGAATAAGGATAAGGCCCTTCTTCAGGATGCAAGATCCTACCTGGAGGAGACCTGCATCAAGTGGCTGCGGCAGTACCTGCAGCACGGGGAGGCAGCGCTGCAGAGCAGT

>Beta1085-E2*01

CTGCCCCCCCCAGAGCATTTCCTGTTGCAGGGGAAGGCTGAGTGTCTCTACACAAATGTGTCCCAGCGGGTGAGGTTTGTGCTGAAGTTCATCTGGGACCAGCAGCAGTACGTTCACTTCGACAGCGATGTTGGCGTGTTTGTGGCTGACACTGTGCTGGGTCAGCCTGATGCCACATACTGGAACAGCCAGAAGGAGGAGTTGGAGTACAGCCGGGGTGCAGTGGACAGCGTCTGCCAGCGCAACTATGGGGTGGCTGAGCAAGCCCATGTGCTTGGCCGC

>Beta1085-E2*02

TGCCCCCCCCCAGAGCATTTCCTGTTGCAGGGGAAGGCTGAGTGTCTCTACACAAATGTGTCCCAGCGGGTGAGGTTTGTGCTGAAGTTCATCTGGGACCAGCAGCAGTACGTTCACTTCGACAGCGATGTTGGCGTGTTTGTGGCTGACACTGTGCTGGGTCAGCCTGATGCCACGTACTGGAACAGCCAGAAGGAGGAGTTGGAGTACAGCCGGGGTGCAGTGGACAGCGTCTGCCAGCGCAACTATGGGGTGGCTGAGCAAGCCCATGTGCTTGGCCGC

>Beta1085-E3*01

GCGAAGCCCAAGGTGAAAGTCTCCCCAGCAAAATCAGGGGCCCAGGCCCACCCGGACGCACTGATTTGCTCCGTGACGGGGTTCTACCCAGGCAACATTGAGGTTAAGTGGTTGAAGAACGGGCAGGAGCAGACGGCCGGAGCGGTGTCCACGGAGCTGATGCAGAACGGAGACTGGACCTTCCAGATCCTGGTGATGCTGGAAGTGACCCTCCACAGCGGGGACATCTACACCTGCCAGGTGGAGCACAGCAGCCTGCCGGGACCCATAACTGTGCTCTGG
